# Total lymphocyte count as a surrogate marker for CD4 count in resource-limited settings

**DOI:** 10.1186/1471-2334-12-128

**Published:** 2012-06-07

**Authors:** Christian Obirikorang, Lawrence Quaye, Isaac Acheampong

**Affiliations:** 1 Department of Molecular Medicine, School of Medical Sciences, College of Health Sciences, Kwame Nkrumah University of Science and Technology (KNUST), Kumasi, Ghana; 2 Department of Medical Laboratory Sciences, School of Medicine and Health Sciences, University for Development Studies, Tamale, Ghana; 3 Laboratory Department, University Hospital, Kwame Nkrumah University of Science and Technology (KNUST), Kumasi, Ghana

**Keywords:** Total lymphocyte count, CD4 count, Sensitivity, Specificity, Human immunodeficiency virus

## Abstract

**Background:**

CD4 testing is the recognized gold standard used to stage HIV/AIDS, guide treatment decisions for HIV-infected persons and evaluate effectiveness of therapy. The need for a less expensive surrogate marker that can be used in resource-limited setting is however necessary. The study sought to assess the suitability of Total lymphocyte count (TLC) as a surrogate marker for CD4 count in resource-limited localities in Ghana.

**Methods:**

This observational study was conducted at the Central Regional Hospital, which has one of the established antiretroviral therapy centres in Ghana. A total of one hundred and eighty-four (184) confirmed HIV I seropositive subjects were included in the study. Blood samples were taken from all the subjects for estimation of CD4 and total lymphocyte counts. The study subjects were further categorised into three (3) groups according to the Centers for Disease Control and Prevention (CDC) classification criteria as follows: CD4 counts (1) ≥ 500 cells/mm^3^ (2) 200–499 cells/mm^3^ and (3) <200 cells/mm^3^. Positive predictive value (PPV), negative predictive value (NPV), sensitivity and specificity of various TLC cut-offs were computed for three groups. Correlation and Receiver Operator Characteristic analysis was performed for the various CD4 counts and their corresponding Total Lymphocyte count obtained.

**Results:**

The sensitivity, specificity, positive and negative predictive values of TLC 1200 cells/ mm^3^ to predict CD4 count were <200 cells/mm^3^ 72.2%, 100%, 100% and 95.7% respectively. A TLC of 1500 cells/ mm^3^ was found to have maximal sensitivity (96.67%), specificity (100%), PPV (100%) and NPV (75.0%) for predicting a CD4 cell count of 200–499 cell/mm^3^. A TLC of 1900 cells/mm^3^ was also found to have a maximal sensitivity (98.45%), specificity (100%), PPV (100%) and NPV (100%) for predicting CD4 count ≥500 cells/mm^3^. A positive correlation was noted between 184 paired CD4 and TLC counts (r = 0.5728).

**Conclusion:**

Total Lymphocyte count can therefore adequately serve as a surrogate marker for CD4 count in HIV patients who are naïve for antiretroviral therapy in resource-limited areas.

## Background

Worldwide estimates of people living with Human Immunodeficiency Virus was approximately 32 million in 2007 with thousands of people getting infected every day [1]. Most people living with HIV are from developing countries with less than 5% receiving antiretroviral therapy [2]. In 2009, an estimated 2.6 million people became infected out of which approximately 1.8 million were from sub-Saharan Africa [2]. The initiation of antiretroviral therapy is based on CD4 counts of less than 350 cells/mm^3^ according to the World Health Organization (WHO) and Centre for Disease Control (CDC). The determination of CD4 count however in resource-limited localities is difficult. A total lymphocyte count (TLC) of <1200 cells/mm^3^ has been recommended in addition to WHO staging (stage II) of the disease, for the initiation of antiretroviral therapy in such localities [3]. The use of absolute lymphocyte count as a marker for HIV progression has been argued in many quarters over the years [4-7]. Studies have suggested that when the absolute lymphocyte count is used in conjunction with blood hemoglobin, it gives a more sensitive marker for HIV progression [6,8] with other studies discrediting the use of TLC in such settings [9,10].

The above disagreements in various study settings necessitated the need for this study to be carried out. The aim of this study was therefore to ascertain existing relationships between CD4 count and TLC and to further ascertain if TLC could be used as a surrogate marker for CD4 counts in the initiation of antiretroviral therapy in resource-limited localities in Ghana.

## Methods

This observational study was conducted at the Central Regional Hospital which is one of the established centres providing anti-retroviral therapy (ART) and located at Cape Coast, the capital of the Central region of Ghana. The study was conducted between August 2007 and May 2008. Blood samples were taken from the subjects before the initiation of ART. This study was approved by the Committee on Human Research, Publications and Ethics (CHRPE), School of Medical Sciences, Kwame Nkrumah University of Science & Technology (KNUST), Kumasi. All patients enrolling in the study completed a written informed consent form in accordance with the Helsinki Declaration. After obtaining consent, demographic questionnaires were completed.

### Total lymphocyte count and CD4 cell count

Blood (5 ml) was drawn into Vacutainer tubes with Ethylenediaminetetraacetic acid (EDTA) and used to determine CD4 cell count and TLC. Samples from the patients were analysed within 2 to 4 hours of collection. TLC was determined by using an automated blood analyzer (CELL-DYN 1800, Abbott Laboratories Diagnostics Division, USA) and CD4 T lymphocytes count was determined using the Becton Dickinson (BD) FASCount system (Becton, Dickinson and Company, Califonia, USA). The BD FASCount system used flow cytometry for the quantification of the CD4 T Lymphocytes.

### Subjects

A total of one hundred and eighty-four (184) HIV I seropositive subjects who were determined by rapid immunochromatographic HIV test kit First Response HIV 1–2 and confirmed with an enzyme-linked immunosorbent assay were included in the study after giving informed consent. The study subjects were categorised into three (3) groups according to the Centers for Disease Control and Prevention Criteria (CDC) classification system that emphasizes the importance of CD4+ T lymphocyte testing in clinical management of HIV-infected persons. The groups are: CD4 counts (1) ≥500 cells/mm^3^; (2) 200–499 cells/mm^3^; and (3) *<*200 cells/mm^3^. Inclusion criteria were at least 18 years of age and HIV-1 seropositivity. Exclusion criteria were antiretroviral therapy and co-morbidity with other medical conditions (e.g. tuberculosis, endocarditis and acute viral infections) which could greatly modify haematologic parameters.

### Statistical analysis

The results were given as mean ± Standard error of mean (SEM). Correlations were evaluated using the Pearson’s correlation test. Sensitivity, specificity, positive and negative predictive values with 95% confidence intervals (CIs) of various cut-off points of the TLC to predict CD4^+^ T-cell count ≥500 cells/mm^3^, 200–499 cells/mm^3^ and < 200 cells/mm^3^ were calculated. For all statistical comparisons, the level of significance was set at *p* < 0.05. Data were analyzed using GraphPad Prism for Windows version 4.02 (GraphPad Software, San Diego, CA, USA).

## Results

**Figure 1:**
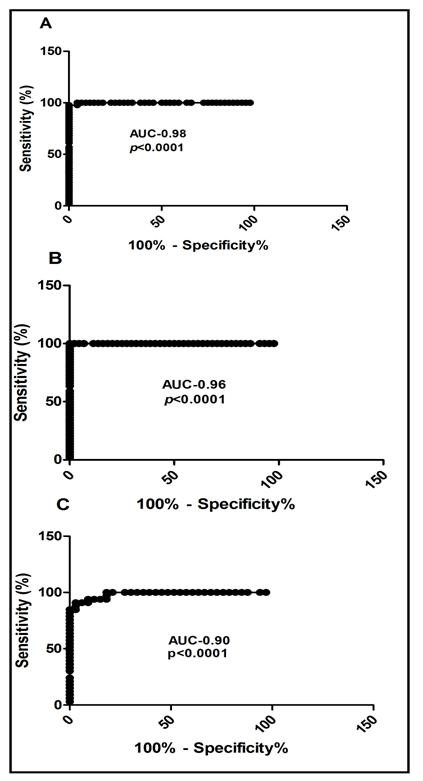
**ROC curve for A. CD4 < 200 cells/mm**^**3**^**, B. CD4 200–499 cells/mm**^**3**^**and C. CD4 >500 cells/mm**^**3**^**.**

**Figure 2:**
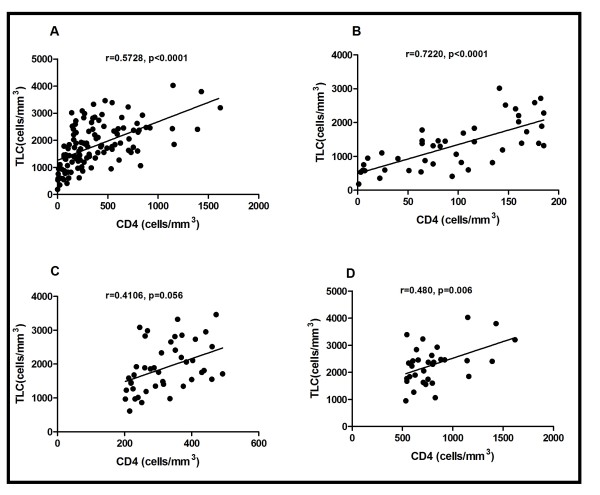
**Correlation between Total Lymphocyte Count and (A) the whole group (B) CD4 < 200 cells/mm**^**3**^**(C) CD4 200–499 cells/mm**^**3**^**and (D) CD4 > 500 cells/mm**^**3**^**.**

The demographic characteristics of the subjects are shown in Table 1. Table 1 shows the means of the ages, CD4 count and TLC of the three groups and these values as expressed as means ± Standard error of the Mean (SEM). Various TLC cut-off, sensitivity, specificity, positive and negative predictive values for the three CD4 groups are shown in Tables 2. Considering the best cut-off values of TLC, that are with the highest sensitivity and specificity combinations, a TLC of 1200cells/mm^3^ was found to have maximal sensitivity of 72.2% and specificity of 100% for predicting a CD4 cell count of < 200 cells/mm^3^. The best TLC cut-off for predicting CD4 count between 200–499 cells/mm^3^ with a maximal sensitivity of 96.67% and specificity of 100% was 1500 cells/mm^3^ and CD4 count ≥500 cells/mm^3^ with maximal sensitivity of 98.45% and specificity of 100% was 1900 cells/mm^3^ as shown in Table 2. Figure 1 shows the ROC curve for the different groups of CD4 counts. The various areas under the curves for the various CD4 groups (AUC) are also shown in Figure 1. The AUC of the various groups were high (closer to 1) making TLC a perfect substitute for CD4 count. A positive Pearson’s correlation coefficient (r) of 0.5728 (*p* < 0.0001) was realised when TLC and CD4 count of the whole group was analysed as shown in Figure 2. A positive correlation (r = 0.7220, p < 0.0001) was demonstrated between TLC and CD4 count for the group with CD4 count <200cells/mm^3^. A positive correlation of (r = 0.4106, p = 0.056) and (r = 0.480, p = 0.006) was also demonstrated for the groups with CD4 between 200–499 cells/mm^3^ and >500 cells/mm^3^ respectively.

**Table 1:** General demographic characteristic of the studied population

	**CD4 count (cells/mm**^**3**^**)**		
	**<200**	**200-499**	**≥500**
***Age*(years)**	37.00 ± 1.61	34.71 ± 1.18	32.79 ± 1.10
**Number of subjects**	71(38.59%)	60 (32.61%)	53 (28.80%)
**Sex**			
***Male***	31 (43.67%)	23 (38.33%)	19 (35.85%)
***Female***	40 (56.33%)	37 (61.67%)	34 (64.15%)
***CD4 (cells/mm***^***3***^***)***	95.95 ± 8.96	325.5 ± 13.26	812.5 ± 47.86
***TLC (cells/mm***^***3***^***)***	1018 ± 105.80	1896 ± 109.00	2292 ± 125.4

The values are expressed as mean ± SEM.

**Table 2:** Sensitivity, Specificity, Positive and Negative predictive values of Total lymphocyte count cut-off for various CD4 groups

	**TLC cut-off (cells/mm3)**	**Sensitivity (%)**	**95% CI**	**Specificity (%)**	**95% CI**	**PPV (%)**	**NPV (%)**
	1000	72.22	46.52 to 90.31	94.44	72.71 to 99.86	100	91.7
**CD4 count <200**	**1200**	**72.22**	**46.52 to 90.31**	**100**	**81.47 to 100.0**	**100**	**95.7**
**(cells/mm**^**3**^**)**	1400	66.67	40.99 to 86.66	100	81.47 to 100.0	77.8	94.1
	1600	55.56	30.76 to 78.47	100	81.47 to 100.0	65.6	91.7
	1000	100	88.4 to 100	66.67	9.4 to 99.2	96.8	100
**CD4 count 200–499**	1200	100	88.4 to 101	67.67	9.4 to 99.3	96.7	66.7
**(cells/mm**^**3**^**)**	**1500**	**96.67**	**82.8 to 99.9**	**100**	**29.2 to 100**	**100**	**75.0**
	1600	93.33	77.9 to 99.2	100	29.2 to 101	100	60.0
	1700	95.45	77.2 to 99.9	63.64	30.8 to 89.1	84.0	87.5
**CD4 count ≥500**	1750	96.45	77.2 to 99.10	72.73	39.0 to 94.0	87.5	88.9
**(cells/mm**^**3**^**)**	1800	97.45	77.2 to 99.11	90.91	58.7 to 99.8	95.5	90.9
	**1900**	**98.45**	**77.2 to 99.12**	**100**	**71.5 to 100**	**100**	**91.7**

TLC-Total lymphocyte count; PPV-Predictive positive value; NPV-Negative predictive value; 95% CI- 95% Confidence interval.

## Discussion

Depletion of lymphocytes, primarily of the CD4 cell subset subsequent to cellular CD4 immunodeficiency has been noted as the hallmark of HIV infection [11,12] and CD4 count has been established as the gold standard for staging HIV/AIDS, guiding treatment decisions for HIV-infected persons and evaluate effectiveness of therapy. Establishing a cut-off value for TLC so as to be used as surrogate marker for CD4 in staging, monitoring and as a guide to treatment decisions in HIV infected persons in resource-limited settings has been argued [10]. But in areas where viral loads and CD4 counts are absent, using the current WHO guidelines which propose the use of TLC in conjunction with clinical data as a criterion for the initiation of ART is the next option [10]. Threshold analysis was done in this study to find the ability of TLC to predict CD4 counts at three different levels thus CD4 < 200 cells/mm^3^, CD4 between 200–499 cells/mm^3^ and CD4 ≥500cells/mm^3^.

The study found that a TLC of 1200 cells/mm^3^ had a maximal sensitivity of 72.2% and a specificity of 100% for a CD4 count <200 cells/mm^3^ with a PPV of 100% and NPV of 95.7%. These maximal sensitivity, specificity, PPV and NPV obtained at this threshold from this study shows a strong relationship between CD4 < 200 cells/mm^3^ and TLC, making the use of TLC as a surrogate marker in remote and deprived areas of Ghana where there is scarcity of laboratory technologies (*i.e.* CD4 equipment not available) a good choice. Total lymphocyte count at a cut-off of 1200 cells/mm^3^ is a good substitute for CD4 < 200cell/mm^3^ in remote and deprived areas of Ghana: thus 3 in 4 individuals would be given the needed medication if a total lymphocyte count of 1200 cells/mm^3^ were used, as recommended by the WHO. This finding is consistent with other reports [5,6,13,14]. Spacek *et al*[6], found sensitivity and specificity of 70.7% and 81.7% with Badri & Wood [13] also finding a sensitivity of 83.4% and a specificity of 87.3%. Kumarasamy *et al*[5] also had similar sensitivity and specificity of 73% and 88% respectively.

However there have been conflicting results that has been reported [10,15,16]. These reports found low sensitivity and specificity between CD4 count and TLC and therefore suggested that TLC could not be used as a surrogate marker.

Daka & Loha [10] found the sensitivity, specificity, positive and negative predictive values of TLC < 1200 cells/mm^3^ to predict CD4 count < 200 cells/mm^3^ to be 41%, 83.5%, 87.9% and 32.5%, respectively. Findings of such studies are obviously conflicting in different countries. These differences could be due to different ethnic, racial, epidemiological and socioeconomic factors [17].

The study was also able to establish different cut-off of TLC for those with CD4 between 200–499 cells/mm^3^ and those with CD4 count ≥500 cells/mm^3^. The maximal cut-off for those with CD4 between 200–499 cells/mm^3^ was 1500 cells/mm^3^ and that of those with CD4 count ≥500 cells/mm^3^ was 1900 cells/mm^3^. This can help in taking decisions as to when to initiate ART in resource-limited setting in Ghana where TLC measurement can easily be done.

From the ROC analysis, the AUC were high for those with CD4 count <200 cells/mm^3^, 200–499 cells/mm^3^ and ≥500 cells/mm^3^ making the use of TLC a good substitute surrogate marker for CD4 count in resource-limited settings.

A positive correlation was established between TLC and CD4 count (r = 0.5728, *p* < 0.0001) of the whole group. There was even a stronger positive correlation in TLC and CD4 counts of those with CD4 <200 cells/mm^3^ (r = 0.7220, *p* < 0.0001). Other authors also obtained a stronger correlation between these parameters. These include Jacobson et al [18], who had r = 0.68; Badri & Wood [13], who also had r = 0.61; and Pascale et al [19], who had r = 0.68.

## Conclusion

The findings suggest that TLC, which is relatively inexpensive and available, is a reasonably accurate tool that can serve as a surrogate marker in HIV patients who are naive to antiretroviral therapy as to when to initiate anti-retroviral therapy in resource-limited settings.

### Limitations

Considering the number of subjects that were enrolled during study period, and the fact that all the subjects were naive for antiretroviral therapy, interpretation of the results obtained should be done with caution.

## Competing interests

We declare that we have no competing interests.

## Authors’ contributions

CO and IA carried out the TLC and CD4^+^ counts, performed the statistical analysis and drafted the manuscript. LQ designed the study and its coordination and participated in the drafting of the manuscript. All authors read and approved the final manuscript.

## Pre-publication history

The pre-publication history for this paper can be accessed here:

http://www.biomedcentral.com/1471-2334/12/128/prepub
